# Time series analysis of in-hospital maternal case fatality ratio in the postpartum period according to pregnancy risks and route of delivery in the regions of Brazil, 2010-2019

**DOI:** 10.1590/S2237-96222022000300011

**Published:** 2022-12-02

**Authors:** Bruna Depieri Michels, Daniela Ferreira D'Agostini Marin, Betine Pinto Moehlecke Iser

**Affiliations:** 1 Universidade do Sul de Santa Catarina, Faculdade de Medicina, Tubarão, SC, Brazil; 2 Universidade do Sul de Santa Catarina, Programa de Pós-Graduação em Ciências da Saúde, Tubarão, SC, Brazil

**Keywords:** Maternal Mortality, Childbirth, Postpartum Period, Time Series Studies

## Abstract

**Objective:**

to analyze in-hospital maternal case fatality ratio in the postpartum period according to pregnancy risks and route of delivery, within the Brazilian National Health System, Brazil and macro-regions, 2010-2019.

**Methods:**

this was an ecological time-series study, using data from the Hospital Information System; in-hospital maternal case fatality ratio in the postpartum period took into consideration maternal hospitalizations with outcome 'death' over the total number of hospitalizations per year, according to pregnancy risks and route of delivery, in the regions.

**Results:**

there were 19,158,167 hospitalizations for childbirth and 5,110 deaths in the period analyzed; maternal case fatality ratio increased from 1.1 (2010) to 1.9 death/10,000 hospitalizations (2019), in usual-risk pregnancies after vaginal deliveries, and decreased from 10.5 (2010) to 7.0 deaths/10,000 hospitalizations (2019) in high-risk pregnancies after cesarean sections; the Midwest region presented the highest and the South region the lowest case fatality ratio for high-risk pregnancies.

**Conclusion:**

in-hospital case fatality ratio was higher for high-risk pregnancies, showing differences according to route of delivery and regions.

**Study contributions**

**Main results**

It could be seen a trend of increased in-hospital maternal case fatality ratio in the postpartum period among usual-risk pregnant women in Brazil, between 2010 and 2019.Regional disparities were observed. High-risk pregnancies presented the highest case fatality ratios.

**Implications for services**

The study shows the importance of attention to pregnancy risks in health systems, so that women can be properly cared, focusing on the impacts of pregnancy risks, including maternal death, in the immediate postpartum period.

**Perspectives**

By identifying differences in the prognosis of usual and high-risk pregnancies, we aimed to improve information to health professionals in order for them to enhance prenatal and immediate postpartum care, directing resources.

## Introduction

Maternal mortality is a public health problem and a serious violation of women's human rights, mainly due to the fact that most cases are characterized as preventable events.^1^

Reducing maternal mortality, regardless of gestational risk, is one of the targets of the World Health Organization (WHO) Sustainable Development Goals for 2030.^2^ In 2019, in Brazil, the maternal mortality ratio (MMR) was 58 maternal deaths per 100,000 live births (LB), a value almost twice as high as the target set by the WHO for 2030, which is 30 deaths per 100,000 LB.^3^

Brazilian obstetric care is based on an interventionist model, which has already led to a "cesarean section epidemic". Currently, Brazil shows the second highest cesarean delivery ratio in the world, having reached the proportion of 55.8% of deliveries that occurred between 2014 and 2017.^4^ However, studies show a higher risk of maternal death after cesarean sections, when compared to the same risk through vaginal deliveries in the country.^5^ ^,^ ^6^

The analysis of information on maternal mortality indicators is essential to know the scenario of women's health and the care provided to them in order to help in decision-making and thus reduce their causes and prevent new deaths. Although the MMR indicator has been widely used, according to the national and international literature, little emphasis is placed on death as an outcome of hospitalizations for childbirth - a fraction of the MMR, related to the immediate puerperium and directly related to care at birth. Within this context, the study aimed to analyze the time series of maternal case fatality ratio in the postpartum period according to pregnancy risks and route of delivery, in Brazil and its macro-regions, within the Brazilian National Health System (*Sistema Único de Saúde* - SUS), between 2010 and 2019.

## Methods

This was an ecological time series study, which analyzed in-hospital maternal case fatality ratio in the postpartum period in Brazil, between 2010 and 2019. According to data from the 2010 Population Census, the total female population of reproductive age (age group 15 to 49 years, according to WHO),^7^ corresponded to 28% (53,669,289) of the Brazilian population. There were 29,157,184 live births registered in the period from 2010 to 2019 in Brazil, 39% of them in the Southeast region, the most populous region in the country.^8^

The study used data from the Hospital Information System of the Brazilian National Health System (*Sistema de Informações Hospitalares do Sistema Único de Saúd*e - SIH/SUS), which records all hospital admissions via the SUS in the national territory, in all five Brazilian macro-regions (North, Northeast, Midwest, Southeast and South), in the period from 2010 to 2019. It is estimated that 80% of childbirths in the country are performed within the SUS.^9^ The SIH/SUS data, made available on the Brazilian National Health System Information Technology Department (*Deparatamento de Informática do Sistema Único de Saúde* - DATASUS) website,^10^ were accessed on December 20, 2020, using the Tabnet application.

The study population was comprised of all pregnant women who were admitted for childbirth in a hospital linked to the SUS, whether public or private. The analysis included hospitalizations for childbirth, identified by Chapter XV - Pregnancy, Childbirth and the Puerperium - of the International Statistical Classification of Diseases and Related Health Problems 10^th^ Revision (ICD-10), and the procedures performed were selected: natural childbirth; natural childbirth in high-risk pregnancy; cesarean delivery; and cesarean delivery in high-risk pregnancy. Pregnancy risk categorization is previously performed by a health professional who was responsible for the care of a pregnant woman. According to the Brazilian Ministry of Health,^1^ high-risk pregnancy involves an individual analysis that takes into consideration previous clinical and reproductive history, in addition to clinical/obstetric events during the current pregnancy, such as obesity with BMI > 40, repeat abortions, fetal growth restriction or death, previous diseases and/or infectious diseases during pregnancy. The data of interest collected for this study were (i) maternal hospitalization for childbirth [based on Hospital Admission Authorizations (*Autorizações de Internação Hospitalar* - AIH) via the SUS]and (ii) maternal deaths after delivery (number of hospital discharges due to death), according to pregnancy risk classification and route of delivery, by Brazilian macro-region, in each year studied.

The outcome variable of interest for the study was death after childbirth, that is, in the immediate puerperium, taking into consideration the number of hospitalizations for childbirth that were discharged due to death. The independent variables analyzed were (i) the year of hospitalization (2010 to 2019), (ii) the region of the country (North, Northeast, Midwest, South, Southeast), (iii) pregnancy risk (high risk, usual risk) and (iv) the route of delivery (vaginal, cesarean section). Pregnancy risk and route of delivery were selected according to the classification: high-risk vaginal delivery, usual-risk vaginal delivery, high-risk cesarean delivery, usual-risk cesarean delivery.

The calculation of in-hospital maternal case fatality ratio in the postpartum period was performed by dividing the number of maternal hospitalizations for childbirth who were discharged from hospital due to death by the total number of maternal hospitalizations for childbirth, multiplied by 10,000, according to the year and region.

In order to analyze the temporal trend of in-hospital maternal case fatality ratio related to the route of delivery and the time/event relationship in the period from 2010-2019, we used Prais-Winten generalized linear regression model,^11^ calculating the coefficient of determination (R2) and the average annual percentage change of values of the series (β coefficient). The response variable (Yi) was in-hospital maternal case fatality ratio in each year; and the explanatory variable (Xi) was the year of death. The value of the positive/negative angular coefficient (β) represents the annual average increase/decrease in maternal case fatality ratio, respectively, for each year analyzed, and it is expressed in percentage points (p.p.) per year. The average in-hospital maternal case fatality ratios, as well as their respective 95% confidence intervals (95%CI) were estimated, aiming to compare the indicators according to the characteristics of interest. The significance level was 5%.

The study project was approved by the Research Ethics Committee of the Universidade do Sul Santa Catarina (CEP Unisul), on December 22, 2020. Opinion No. 4,482,150. We used data in the public domain, and it is not linked to individual data.

## Results

In Brazil, the total number of pregnant women hospitalized for childbirth between 2010 and 2019 was 19,158,167; 5,110 deaths following childbirth were recorded in the same period. There were 17,137,656 hospitalizations for childbirth classified as usual-risk pregnancy, and 2,020,511 hospitalizations (approximately 12%) of high-risk pregnant women, according to data showed in Table 1.

**Table 1:** Number of hospitalizations and deaths following the postpartum period recorded in the Hospital Information System of the Brazilian National Health System (SIH/SUS), according to pregnancy risks and route of delivery, by macro-regions, Brazil, 2010-2019

Brazil and macro-regions	Usual risk						High-risk pregnancy				
	Vaginal delivery			Cesarean section delivery			Vaginal delivery			Cesarean section delivery	
	Hospitalizations	Deaths		Hospitalizations	Deaths		Hospitalizations	Deaths		Hospitalizations	Deaths
Brazil	10,618,496	1,522		6,519,160	2,101		792,592	378		1,227,919	1,109
North	1,345,161	310		800,707	325		53,320	43		101,225	114
Northeast	3,543,874	540		2,053,375	604		306,758	186		435,899	465
Southeast	319,167	117		2,183,128	702		319,167	117		509,262	415
South	88,894	16		916,743	285		88,894	16		125,403	49
Midwest	24,417	16		565,207	185		24,417	16		56,130	66

**Figure 1:**
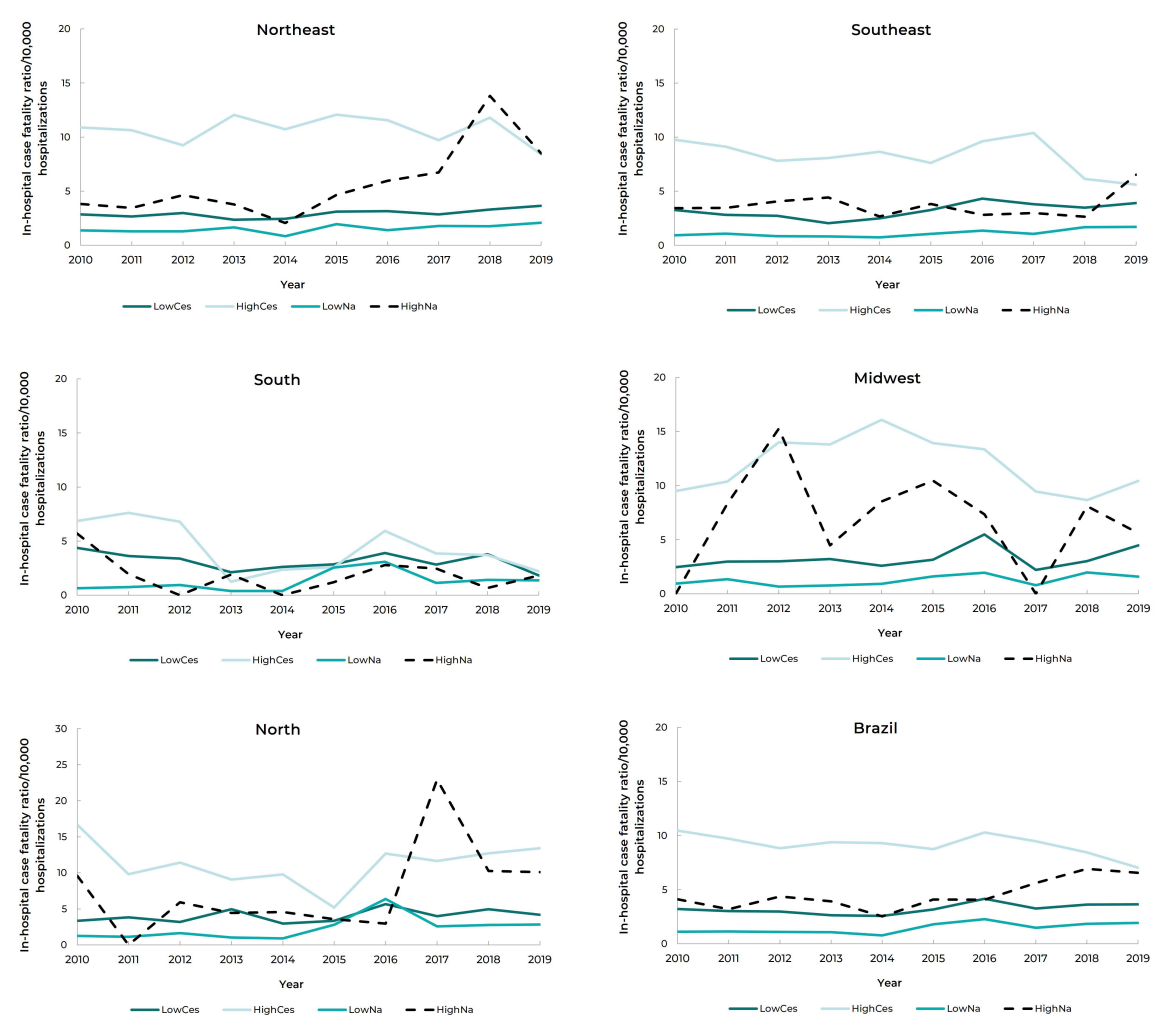
Temporal trend of in-hospital maternal case fatality ratio in postpartum period according to pregnancy risks and route of delivery, by macro-regions, Brazil, 2010-2019

Legend: LowCes: Cesarean sections in usual-risk pregnancies; HighCes: Cesarean sections in high-risk pregnancies; LowNa: Natural childbirth in usual-risk pregnancies; HighNa: Natural childbirth in high-risk pregnancies.

In Brazil, in-hospital case fatality ratio among usual-risk pregnant women after cesarean sections was 3.2 deaths per 10,000 in 2010 and reached 3.6 per 10,000 in 2019, indicating stability and an average of 3.2 (95%CI 2.9;3.6) deaths per 10,000, during the period. The North region was the only region to show a significant increase in in-hospital case fatality ratio after childbirth via cesarean section in usual-risk pregnancy, rising from 3.4 deaths per 10,000 hospitalizations for childbirth in 2010 to 4.2 deaths per 10,000 hospitalizations in 2019 (p-value = 0.003). Taking into consideration the usual-risk vaginal deliveries performed in the country as a whole, case fatality ratio increased significantly, from 1.1 death per 10,000 hospitalizations for childbirth in 2010 to 1.9 in 2019 (p-value = 0.001), as described in Table 2 and Figure 1.

**Table 2:** In-hospital maternal case fatality ratios (per 10,000 hospitalizations) in the postpartum period in usual-risk pregnancies during hospitalization, by period, according to the route of delivery and the macro-regions, Brazil, 2010-2019

Year	Usual-risk pregnancies																
	Brazil			North			Northeast			Southeast			South			Midwest	
	Cesarean section	Vaginal delivery		Cesarean section	Vaginal delivery		Cesarean section	Vaginal delivery		Cesarean section	Vaginal delivery		Cesarean section	Vaginal delivery		Cesarean section	Vaginal delivery
2010	3.2	1.1		3.4	1.3		2.9	1.4		3.3	0.9		4.4	0.7		2.5	1.0
2011	3.0	1.1		3.8	1.1		2.7	1.3		2.8	1.1		3.6	0.7		3.0	1.4
2012	3.0	1.1		3.2	1.7		3.0	1.3		2.7	0.9		3.4	0.9		3.0	0.7
2013	2.6	1.1		5.0	1.0		2.4	1.7		2.1	0.8		2.1	0.4		3.2	0.8
2014	2.6	0.8		3.0	0.9		2.5	0.8		2.5	0.7		2.6	0.4		2.6	0.9
2015	3.2	1.8		3.3	2.8		3.1	2.0		3.3	1.1		2.9	2.5		3.1	1.6
2016	4.2	2.3		5.7	6.4		3.2	1.4		4.3	1.4		3.9	3.1		5.5	2.0
2017	3.3	1.5		4.0	2.6		2.9	1.8		3.8	1.1		2.8	1.1		2.2	0.8
2018	3.6	1.8		5.0	2.8		3.3	1.8		3.5	1.7		3.8	1.4		3.0	2.0
2019	3.6	1.9		4.2	2.8		3.7	2.1		3.9	1.7		1.8	1.4		4.5	1.6
Average	3.2	1.5		4.0	2.3		2.9	1.5		3.2	1.1		3.1	1.3		3.3	1.3
95%CI^a^	2.9;3.6	1.1;1.8		3.4;4.7	1.2;3.5		2.7;3.2	1.3;1.8		2.7;3.7	0.9;1.4		2.6;5.9	0.6;1.9		2.6;3.9	0.9;1.6
β^b^	0.08	0.11		0.14	0.28		0.09	0.08		0.10	0.08		-0.45	0.12		0.12	0.09
p-value	0.100	0.001		0.003	0.040		0.060	< 0.001		0.238	0.040		0.020	0.112		0.009	0.021

a) 95%CI: 95% confidence interval; b) β: Beta coefficient of regression, indicating annual percentage change (APC).

Regarding usual-risk pregnancies, the North, Northeast, Midwest and Southeast regions followed the trend of increased in-hospital case fatality ratio after vaginal deliveries, at the national level, during the study period. In the North region, it could be seen an increase in case fatality ratios from this route of delivery, rising from 1.3 death per 10,000 hospitalizations in 2010 to 2.8 per 10,000 in 2019 (average of 2.3 in the period - 95%CI 1.2;3.5), while in the Northeast region, it rose from 1.4 (2010) to 2.1 (2019) and the average was 1.5 - 95%CI 1.3;1.8. In the Midwest region, there was an increase in maternal case fatality ratios , from 1.0 per 10,000 hospitalizations for childbirth (2010) to 1.6 (2019), and an average of 1.3 (95%CI 0.9;1.6) per 10,000. In the Southeast region, the ratio rose from 0.9 in 2010, to 1.7 in 2019, with an average of 1.1 (95%CI 0.9;1.4). In the South region, which had already showed lower indicators, in-hospital case fatality ratio remained stable throughout the period.

With regard to high-risk pregnancies, case fatality ratio after cesarean sections in Brazil, which was 10.5 deaths per 10,000 hospitalizations for childbirth, in 2010 decreased to 7.0 per 10,000 in 2019, with an average of 9.2 (95%CI 8.5;9.9) in the period (Table 3). 

**Table 3:** In-hospital maternal case fatality ratios (per 10,000 hospitalizations) in the postpartum period in high-risk pregnancies during hospitalization, by period, according to the route of delivery and the macro-regions, Brazil, 2010-2019

Year	Usual-risk pregnancies																
	Brazil			North			Northeast			Southeast			South			Midwest	
	Cesarean section	Vaginal delivery		Cesarean section	Vaginal delivery		Cesarean section	Vaginal delivery		Cesarean section	Vaginal delivery		Cesarean section	Vaginal delivery		Cesarean section	Vaginal delivery
2010	10.5	4.1		16.7	9.6		10.9	3.8		9.8	3.5		6.9	5.7		9.5	0.0
2011	9.7	3.2		9.8	0.0		10.6	3.5		9.1	3.5		7.6	2.0		10.4	8.3
2012	8.8	4.4		11.4	5.9		9.2	4.6		7.8	4.1		6.8	0.0		14.0	15.3
2013	9.4	3.9		9.1	4.4		12.1	3.8		8.1	4.4		1.2	1.9		13.8	4.5
2014	9.3	2.5		9.8	4.6		10.7	2.1		8.6	2.7		2.4	0.0		16.1	8.5
2015	8.7	4.1		5.2	3.6		12.1	4.7		7.6	3.8		2.6	1.2		13.9	10.5
2016	10.3	4.1		12.7	3.0		11.6	6.0		9.6	2.8		6.0	2.8		13.4	7.3
2017	9.5	5.6		11.7	22.9		9.7	6.7		10.4	3.0		3.9	2.5		9.5	0.0
2018	8.4	6.9		12.7	10.3		11.8	13.8		6.1	2.7		3.7	0.7		8.7	8.1
2019	7.0	6.6		13.4	10.1		8.4	8.5		5.6	6.5		2.2	1.8		10.4	5.6
Average	9.2	4.5		11.2	7.4		10.7	5.7		8.3	3.7		4.3	1.9		12.0	6.8
95%CI^a^	8.5;9.9	3.5;5.5		9.1;13.4	2.9;12.0		9.8;11.6	3.3;8.2		7.2;9.4	2.9;4.5		2.7;5.9	0.7;3.0		10.1;13.8	3.5;10.1
β^b^	-0.29	0.31		-0.09	0.99		-0.06	0.78		-0.32	0.13		-0.45	-0.27		0.08	-0.07
p-value	0.004	0.050		0.853	0.122		0.680	0.048		0.160	0.558		0.018	0.403		0.878	0.915

a) 95%CI: 95% confidence interval; b) β: Beta coefficient of regression, indicating annual percentage change (APC).

Despite the reduction, the highest case fatality ratio after childbirth in the period, in Brazil and in all its regions, remains for high-risk pregnancies and after cesarean section delivery (Table 3 and Figure 1).

Regarding the macro-regions, only the South region showed a significant reduction in in-hospital maternal case fatality ratios in the postpartum period, after cesarean section for high-risk pregnancies, from 6.9 deaths per 10,000 hospitalizations in 2010 to 2.2 in 2019 (p-value = 0.018). In the Midwest region, it could be seen the highest in-hospital case fatality ratio among women who faced a high-risk pregnancy, especially after cesarean sections: 12.0 (95%CI 10.1;13.8), an average higher than the national and those of the Southeast and South regions. By the way, the Southern region presented the lowest in-hospital case fatality ratios for high-risk pregnancies in Brazil, either after cesarean sections (4.3; 95%CI 2.7;5.9), or after vaginal delivery (1.9; 95%CI 0.7;3.0).

With regard to in-hospital case fatality ratio after high-risk vaginal deliveries, the national ratio was 4.1 deaths per 10,000 hospitalizations in 2010 and 6.6 in 2019, with an average of 4.5 (95%CI 3.5;5.5) over the period. Only the Northeast region showed an increasing trend, rising from 3.8 (2010) to 8.5 deaths per 10,000 hospitalizations for childbirth (2019), with an average ratio of 5.7 (95%CI 3.3;8.2).

When comparing the average in-hospital case fatality ratio in the postpartum period by pregnancy risk in Brazil, between 2010 and 2019, it was higher in high-risk pregnant women after cesarean section (9.2; 95%CI 8.5;9.9) and vaginal delivery (4.5; 95%CI 3.5;5.5), followed by usual-risk pregnancies after cesarean sections (3.2; 95%CI 2.9;3.6); and the lowest in-hospital maternal case fatality ratio was identified after vaginal delivery in usual-risk pregnant women (1.5; 95%CI 1.1;1.8). This national pattern was similar to that found in the Northeast, Southeast and Midwest regions, where average case fatality ratios ranged from 2.9 to 3.3 deaths per 10,000 hospitalizations for cesarean delivery, and from 1.1 to 1.5 death per 10,000 hospitalizations for vaginal delivery among usual-risk pregnant women; in these same regions, for high-risk pregnancy deliveries, the average case fatality ratios ranged from 8.3 to 12.0 deaths per 10,000 hospitalizations for cesarean delivery, and from 3.7 to 6.8 deaths per 10,000 hospitalizations for vaginal delivery. In the South region, in turn, in-hospital case fatality ratios for high-risk pregnant women who underwent cesarean section presented the highest case fatality ratio in the postpartum period (4.3; 95%CI 2.7;5.9), followed - in the same South region - by in-hospital case fatality ratios for usual-risk pregnant women and cesarean delivery (3.1; 95%CI 2.6;5.9). In the South region, the highest case fatality ratio occurred after cesarean deliveries, regardless of pregnancy risk, and the lowest ratios, after vaginal delivery, also without significant differences according to pregnancy risk. In the North region, there was a statistically significant difference only for deaths after high-risk cesarean sections (11.2; 95%CI 9.1;13.4), whose case fatality ratio was higher when compared to case fatality ratio after usual-risk vaginal and cesarean deliveries.

## Discussion

In this study, it could be seen an increasing trend in in-hospital case fatality ratio after vaginal delivery in usual-risk pregnant women, in addition to a reduction in case fatality ratio after cesarean delivery in high-risk pregnant women, in Brazil, between 2010 and 2019. High-risk pregnancies showed the highest case fatality ratios in the country, regardless of the route of delivery. The analysis according to the macro-regions showed that the South region was the only national region to present a reduction in in-hospital maternal case fatality ratio among high-risk pregnant women who underwent cesarean section.

The increasing trend in maternal case fatality ratio after childbirth in usual-risk pregnancies, found in the study, was not in agreement with maternal mortality data published in the most recent epidemiological bulletin of the Ministry of Health,^3^ which takes into consideration deaths during pregnancy and within 42 days after delivery. By consulting the aforementioned bulletin, it could be seen a decreasing trend in maternal mortality between 1990 and 2019, with a reduction in the ratio of decline as of 2001. It can be suggested that, despite the reduction in mortality during pregnancy and late puerperium (up to 42 days), there was an increase in the number of maternal deaths soon after delivery, still during hospital stay, characterizing in-hospital case fatality ratio in the postpartum period evaluated in this study. The first days of the puerperium correspond to the period when the highest number of postpartum hemorrhages occurs, which has been the leading cause of maternal death in the world in the last 25 years.^12^ Deaths due to postpartum hemorrhage are strongly related to problems in management of obstetric hemorrhage and organizational/structural dysfunctions in the maternity hospital that provides care to the pregnant woman, contributing to the delay in the management of postpartum bleeding. The importance of the incorporation of protocols that prevent postpartum hemorrhage in Brazil is evidenced, as a way to reduce maternal death in the early puerperium.

However, it is noteworthy that, in order to achieve the goal of reducing maternal mortality agreed upon by the Millennium Development Goals (MDGs) by 2015, the Ministry of Health, in 2008, intensified the surveillance of maternal death in order to improve information on the causes of death among puerperal women.^14^ Guidelines were defined, such as decentralization of death surveillance actions and the integrated and articulated action of surveillance and assistance of the three management levels of the SUS.^15^ Based on the investigation and use of correction factors, Brazil showed an increase in deaths among women of reproductive age of around 26% in 2009 and 29% in 2017.^14^ It is worth highlighting that an increase in the number of deaths in the period may be influenced by improvement in registration and not only by worse maternal outcomes. Nevertheless, the country did not reach the target of reducing maternal mortality agreed for 2015, reaching a much higher value (62 maternal deaths per 100,000 LBs) than that stipulated in the MDGs, which is 35.8 per 100,000 LBs.^14^

Unlike usual-risk pregnancies in Brazil, deaths related to high-risk pregnancies presented stability in the study period, taking into consideration vaginal deliveries; and reduction, taking into consideration cesarean deliveries. In agreement with the observed data, the Ministry of Health published a statement reporting that prenatal and postpartum care offered by the SUS has led to a reduction in the number of maternal deaths due to hypertension, hemorrhage and infectious syndromes, pathologies often related to high-risk pregnancies.^16^

In addition, only the South region showed a reduction in in-hospital case fatality ratio in a subgroup studied, within the high-risk cesarean section group. The three Southern states are among the five states with the highest human development index (HDI) in the country,^17^ a fact that may have contributed to better childbirth and postpartum care in that region. In line with this finding, a study conducted in Switzerland, a country with the 3^rd^ best HDI in the world,^18^ also found a reduction in mortality after cesarean sections between 2005 and 2014.^19^

It could be seen significant differences among the macro-regions in this study. In the Northeast region, there was an increase in case fatality ratio among hospitalizations of pregnant women who delivered vaginally, regardless of pregnancy risk, while in the South region, there was a reduction in case fatality ratio among high-risk pregnant women who underwent cesarean section. A study evaluating maternal mortality ratio (MMR) conducted in Brazil, between 1997 and 2012, found an increase in MMR in the Northeast region and a decrease in the South region during those years.^20^ Within this context, it is suggested that socioeconomic differences and quality of childbirth care among the regions are important influencers of maternal mortality. The Northeast region has the highest percentage of illiteracy^21^ among the five regions and their municipalities, some of the worst ratios of socioeconomic vulnerability in the country,^22^ in addition, least developed regions tend to have greater difficulty in accessing and using health services.^23^

The highest case fatality ratios in the postpartum period occurred after cesarean section in high-risk pregnancies. When it comes to pregnancy risk, the Ministry of Health considers the consequences of high-risk pregnancy as some of the leading causes of maternal death in the world.^1^ In fact, high-risk pregnancies increase caesarean section ratios, especially in cases of imminent risk of maternal death. However, it is worth mentioning that high-risk pregnancy is not an absolute indication for cesarean section and therefore, the health professional in charge of attending the delivery should previously evaluate the woman’s condition. Depending on each case, it is possible to wait for the spontaneous onset of labor or perform induction of labor.^24^

High-risk pregnant women presented a higher in-hospital case fatality ratio in the postpartum period than usual-risk pregnant women, regardless of the route of delivery. Despite the indication for more frequent monitoring during high-risk prenatal care,^1^ it is assumed that not all conditions are adequately controlled, with an increased risk of complications and deaths remaining for women with pre-existing conditions. Only the South region showed a different pattern from that of Brazil: in that region, usual-risk pregnant women undergoing cesarean section had the second highest case fatality ratio in the postpartum period. Prenatal care provided in the South region of the country is considered three times more accessible, when compared to the same service provided in other Brazilian regions, and presents the highest levels of guidance for pregnant women about gestational risks.^25^ Thus, it is suggested that in the southernmost region of Brazil, the highest quality of prenatal care, together with the highest population education, may have made it easier to control comorbidities among pregnant women, reducing deaths due to diseases and highlighting deaths resulting from surgical procedures.

This study also showed that, for women, there was a higher risk of death when the route of delivery was cesarean section, in comparison with vaginal delivery, regardless of pregnancy risk. However, it is noteworthy that the highest occurrence of death after cesarean section may be associated with the indication for surgical delivery and not directly to the procedure, given that conditions of acute maternal-fetal distress may determine the choice of cesarean section as the fastest way to resolve that condition.^1^ However, cesarean delivery increases the risk of surgical infection by five times, and sepsis is one of the leading causes of maternal death in the world, increasing the risk of death by 3.5 times.^26^ A Brazilian cohort has also associated excess cesarean sections with a higher risk of unfavorable outcomes for puerperal women: 56% higher risk of early complications, 79% higher risk of urinary tract infection, and 2.98 times as likely of postpartum infection.^5^ A systematic review conducted in Latin America found a higher risk of death after cesarean sections, when compared to vaginal delivery [odds ratio (OR) from 1.6 to 7.08].^6^ In Brazil, the popularization of cesarean section makes surgical delivery in women at usual obstetric risk reach more than 45.5%.^27^ Cesarean section is an important option in obstetrics, especially in conditions of obstetric emergencies that require a rapid resolution for pregnancy, and in conditions in which vaginal delivery is contraindicated; however, as it is a surgical procedure and given the risk of complications, cesarean delivery should be performed under appropriate pathological indication.^28^

As limitations of this study, we should mention those related to the use of secondary data, influenced by the quality of the record made by the health professional who provided care, when characterizing and recording the pregnancy risk and the route of delivery. Moreover, because this is an ecological study, there is an exploratory purpose based on aggregated data, without a cause-effect relationship being defined. It is worth highlighting that the deaths of women during hospitalization for childbirth were evaluated, that is, at the hospital level. Thus, the study does not reflect all maternal deaths due to labor, especially those performed in an unsafe environment and/or without adequate medical care, although these represent about 0.6% of deliveries.^29^

It is important to highlight that the maternal deaths that occurred after childbirth and only during hospital stay were evaluated, we did not include the 42 days postpartum evaluated in the WHO maternal mortality indicator. Furthermore, maternal deaths due to abortion or other causes that were not recorded in the system as resulting from hospitalization for childbirth were not taken into consideration, and we did not evaluate specific data from the Mortality Information System (*Sistema de Informações sobre Mortalidade* - SIM). Thus, the data showed here are not fully comparable to the MMR which, in fact, is further explored in the scientific literature, limiting the relationship with evidence on the subject. Finally, the low number of publications on maternal case fatality ratio due to childbirth according to pregnancy risk strengthens the contribution of this study to the understanding of women's health care during childbirth. In order to improve the data on maternal death in the immediate postpartum period, it is suggested, as a next step, the calculation of mortality ratios by linking SIH/SUS data with those from other health information systems, such as SIM and the Live Birth Information System (*Sistema de Informações sobre Nascidos Vivos* - SINASC).

This study shows a trend of increased in-hospital case fatality ratio in the postpartum period among usual-risk pregnant women who delivered vaginally in Brazil between 2010 and 2019. Regional disparities were observed, mainly in the South region, when compared to the other regions. High-risk pregnancies presented the highest case fatality ratios in Brazil, with the highest ratio occurring after cesarean sections.
